# Tracking microplastics at the source: a comparative study of fluorescent and FTIR microscopy at a drinking water intake in the Perak River, Malaysia

**DOI:** 10.1007/s10661-025-14127-x

**Published:** 2025-05-30

**Authors:** Irfan Hassan, Sumathi Sethupathi, Mohammad J. K. Bashir, Abdul Latif Ahmad, Purushothaman Parthasarathy

**Affiliations:** 1 Faculty of Engineering and Green Technology, Universiti Tunku Abdul Rahman, 31900 Kampar, Perak Malaysia; 2 School of Engineering and Technology, Central Queensland University, 120 Spencer St, Melbourne, VIC 3000 Australia; 3 School of Chemical Engineering, Engineering Campus, Universiti Sains Malaysia, Seri Ampangan, 14300 Nibong Tebal, Pulau Pinang Malaysia; 4 Department of Civil Engineering, College of Engineering and Technology, SRM Institute of Science and Technology, Kattankulathur, Chengalpattu, 603203 Tamilnadu India

**Keywords:** River water, Microplastics characteristics, Drinking water abstraction point, FTIR microscopy, Fluorescence microscopy, Polymer

## Abstract

Asian rivers are significant carriers of microplastics, posing major environmental and health risks. It is critical to understand the quantity and characteristics of MPs in freshwater, particularly river water used as a source of drinking water. Microplastic pollution in freshwater is a serious issue, but there is surprisingly little data on it, especially in Malaysia. This study aims to investigate the presence and characteristics of microplastics in Perak River water, specifically at a water treatment plant point of abstraction, to understand the quantity and features of MPs in river water used as a source of drinking water, and to inform mitigation measures to reduce riverine MP contamination. Water samples were collected in February and July 2023 from the point of abstraction near the Perak River in Malaysia. We analyzed microplastics based on size, shape, and polymer type using fluorescence and FTIR microscopy. Fluorescence microscopy detected more particles on average, while FTIR microscopy identified 12 distinct polymer and semi-synthetic polymers, with Rayon and PE dominating. Most MPs’ particle sizes ranged from 1 to 10 µm. MPs in the shape of granules and irregulars were often discovered. Study finds FTIR microscopy a more suitable and accurate method for identifying MPs compared to fluorescence microscopy. Numerous unidentified particles were also detected using FTIR and fluorescence microscopy. This research helps authorities to develop mitigation measures to reduce riverine MP contamination in drinking water.

**Graphical abstract:**

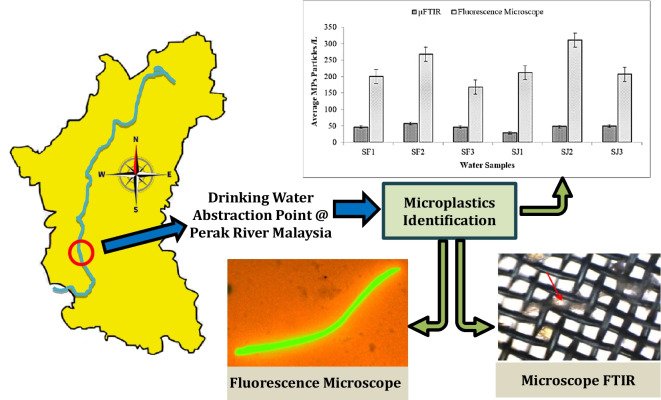

## Introduction

The world is facing a huge problem with plastic pollution, and Malaysia is one of the countries contributing to this issue. Every year, 413.8 million tons of plastic waste are generated globally, but only a small amount is recycled. As a result, a large amount of plastic ends up in the environment, breaking down into tiny pieces called microplastics (MPs) (Global Plastic Production Statista 2023; Horton et al., 2017). MPs refer to plastic particles less than 5 mm but greater than 1 µm (Idehara et al., 2025). They can originate from various sources, such as the breakdown of bigger plastic objects, microbeads in personal care products, and synthetic fibers from garments. Nanoplastics are tiny plastic particles, measuring between 1 nm and 1 µm (Padha et al., 2025). They can be generated by the breakdown of bigger plastic particles or purposely created for use in certain goods (Horton & Dixon, 2018; Horton et al., 2017).

The existence of MPs in freshwater has grown to be a major problem over the past few decades (Alazaiza et al., 2024). 2.4 million tons of plastic debris might enter the ocean each year via the riverine system pathway (Anuar et al., 2023). Recently, Malaysia has been listed as one of the ocean plastic waste polluters (Lugas Wicaksono, 2023). It is estimated that over 0.94 million tons of inappropriate disposal of plastic garbage are disposed of inappropriately globally every day. Moreover, reports state that Malaysia emits a substantial amount of plastic waste, ranging from 0.14 to 0.37 million tons each year, which finds its way to the oceans and various inland freshwater ecosystems, including rivers, lakes, ponds, wetlands, streams, and springs (Jambeck et al., 2015). This occurs due to a variety of factors, including atmospheric deposition, landfills, wastewater treatment plants, agricultural runoff, and fisheries operations, affecting severe pollution of aquatic life (Choong et al., 2021; Li et al., 2020; Wong et al., 2020). Most of these plastics end up as MPs.

Rivers are vital to Malaysia, providing 97% of the country’s drinking water and supporting industrial activities. MP pollution poses a major risk to human health. There is evidence from reports that MPs are discovered in blood clots in the heart, brain, and legs (Liang et al., 2024). Consequently, it is essential to evaluate the amounts of MPs in waterways; so far, only a small number of rivers in Malaysia have been analyzed for MP pollution. Table 1 shows the details of the research work conducted in Malaysia’s river water on MPs up to September 2023.

**Table 1:** MP abundance in freshwaters

Location	Sample	Abundance	Type of polymer	Size	Shape	Color	Ref
Kelantan River, Kelantan	Water	Average of 179.6 items/L	PTFE, PE, and rayon	< 300–5000 μm	Fibers, fragments, films, pellets, and foam	Transparent, blue, red, black, white, purple, yellow, brown, green, and orange	Anuar et al. (2023)
Langat River, Kelantan	Water	Average of 1464.8 items/L	PE, PP, PET, polyolefin, and rayon				
Kemena River, Sarawak	Water	From 60 to 128 items/L (mean 94 ± 34)	PE, PS, PC, and PET	0.1–1 mm	Fibers, films, foam and fragments	Blue and transparent	Karing et al. (2023)
	Sediment	21 to 40 (mean 28 ± 10.44)					
Niah River, Sarawak	Water	46 to 76 items/L (mean 56 ± 17.32)	Not mentioned				
	Sediment	45 to 125 items/kg (mean 76.67 ± 42.52)					
Melayu River, Johor	Water	0–6 particles/L water samples	PE, PET	0.35–3410 µm	Film and fiber	Blue, red, green, transparent, and black	Primus and Azman, (2022)
Sungai Dungun, Terengganu, Malaysia	Water	38.7–300.8 item/m^3^	Rayon, PP, PAN	200 µm	Fragment, fiber	Transparent, black, blue, red, green, brown, purple, white	Yang et al. (2020)
Cherating River, Pahang	Water	Average 0.0042 ± 0.0033 particles/m^3^	Not mentioned	< 0.1–5 mm	Line, fragment, film, foam	Transparent, black, white	Pariatamby et al. (2020)
Skudai River, Johor	Sediment	200 ± 80 particles per kg	Not mentioned	100–5000 μm	Fragment, line/fiber, and film	Blue, transparent, yellow, red, and white	Sarijan et al. (2018)
Tebrau River, Johor	Sediment	680 ± 140 particles per kg		< 100 to 5000 μm			

Researchers in Malaysia have discovered that a combination of primary and secondary microplastic sources contributes to the occurrence of MPs in river water systems (Choong et al., 2021). Four studies have been reported on the types of polymers, using Fourier-transform infrared spectroscopy microscope (µFTIR) and attenuated total reflectance-FTIR (ATR-FTIR) techniques to identify and characterize the MPs. The others were only using microscopy techniques and thus did not report the type of polymers. The number of particles or items and the size of MPs varied tremendously among the works reported in Table 1. The source of the MP particles in these rivers was concluded to be local human activities, wastewater treatment plants, and industrial pressure (Sarijan et al., 2018). Both water and sediment samples revealed that the most common microplastic shapes were fibers, fragments, and films, resulting from the degradation of larger plastic debris rather than originating from microbeads in cosmetics. Since fishing is the primary driver of the local economy, fibers may come from the use of fishing equipment. Fibers may originate from household wastewater because the research region is close to residential areas (Primus & Azman, 2022). Different colors were also identified, with blue and transparent being common among the studies. The colors were identified based on particles that have a size of more than 0.1 mm. For MPS particle sizes of less than 0.1 mm, color was not mentioned in any of the studies summarized in Table 1. Comparing one of the latest reports from the U-Taphao River in Songkhla Province, Thailand, there is a lower exposure of MPs compared to the ones reported in Malaysian rivers. The average occurrence of MPs in the Malaysian River was reduced from upstream to downstream, i.e., from 0.66 ± 0.09 to 0.11 ± 0.02 particles/L. Most of them were fiber, and the predominant size ranges were 500–1000 µm. The types of MPs identified were polyethylene (PE), polypropylene (PP), rayon, polyethylene terephthalate (PET), nylon, and poly (ethylene: propylene). Blue-colored particles were reported the most by Chinfak et al. (2021).

Perak’s rivers remain unevaluated for microplastics, despite the state’s significant river basins. Global microplastic studies typically focus on river water and sediment samples from various sections, including upstream, midstream, and downstream areas, as well as drinking water sources. Thus far, no report has been found on the drinking water point of abstraction from the river for the water treatment plant in Malaysia. Hence, this study will be among the first research projects to create a database of MP abundance and characterization in the water from Perak rivers that are used for drinking purposes after water treatment. This information shall provide baseline information to policymakers and governmental water bodies in Malaysia, as well as globally, for a better understanding of MP occurrence in river water, which is a source point for drinking water treatment plants. This study was conducted to detect the abundance of MPs in the Perak River, especially at the point of the inlet to an active drinking water treatment plant. This study employed two different methods, fluorescent microscopy and FTIR, to identify and characterize microplastics (MPs), comparing their reporting accuracy.

## Materials and methods

### Study area

**Fig. 1:**
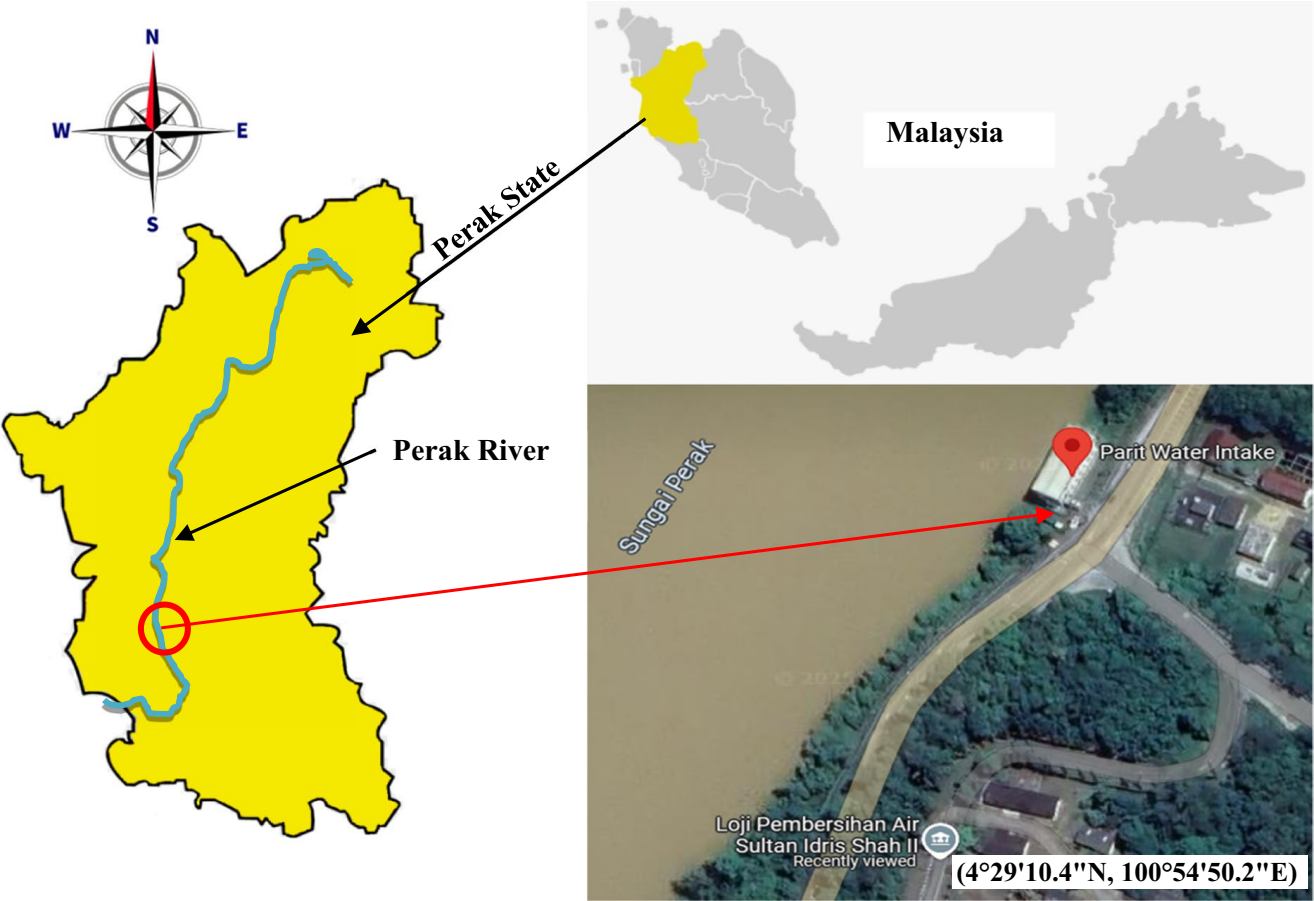
Map of Malaysia with a highlight on the Perak State, Perak River, and water collection site (4°29'10.4"N, 100°54'50.2"E)

This study was conducted at the Perak River in Malaysia, the second-longest river in Peninsular Malaysia, stretching 427 km. The Perak River basin covers an extensive catchment area of 15,151 km^2^, making it the largest in the region, encompassing approximately 70% of the state’s surface area (Ahmad et al., 2017). A visual representation of the Perak River is provided in Fig. 1, displaying a Google image of the river (Google, 2024).

The Perak River is unique among Malaysian rivers in that it has four dams constructed on its main course, creating reservoirs that stretch from North to South (Ismail et al., 2018). The quality of Perak River water is classified as Class II. The water quality class (Class I being the greatest and Class V being the worst) was established using the Water Quality Index (WQI) (Salam et al., 2020, 2019a, 2019b). The water from this river basin has been used for drinking water production, agricultural activities, recreation, and other activities.

### Sample collection

River water samples were collected manually from the Parit Water Intake facility (4°29′10.4″N, 100°54′50.2″E). This facility feeds water into the Water Treatment Plant via an intake system, with the well’s base 1.5 m below “low water level,” operating at an average flow rate of 0.694 cubic meters per second. The facility is designed to filter out debris larger than 5 mm before feeding into the water treatment plant. Water samples were collected in February and July 2023. There were three samples for each month, labeled as SF1, SF2, and SF3 for samples collected in February and SJ1, SJ2, and SJ3 for samples collected in July. Samples were drawn from the filtered tank using a 5-L stainless-steel bucket. Fifteen liters of water samples were collected per sampling session and stored in three 5-L glass bottles. To prevent contamination and ensure the accuracy of the results, the stainless-steel bucket and glass bottles used for sample collection were thoroughly rinsed with 10% hydrochloric acid. After collecting, the samples were stored in the laboratory fridge at a temperature of 4 °C before undergoing further analysis (Karing et al., 2023) (APHA, 1992).

### Quality assurance and control

The laboratory setting was critical for microplastic analysis. Control studies were performed, and no plastic contaminations were found in the blank samples. Deionized water was filtered and exposed to the environment during sample analysis before being re-filtered after processing. All glassware was thoroughly cleaned with ultra-pure water, and plastic equipment was kept to a minimum. The study was carried out in a restricted, windowless room with no polymer-based goods, and lab coats were made of 100% cotton. Filtration equipment and glass containers were carefully cleaned and wrapped in aluminum foil before and after each test. Blank samples were tested in parallel to assess airborne contamination.

### Extraction of microplastics

The sample was prepared by adding a 5.00 M sodium chloride (NaCl) solution, which was created by dissolving 292 g of NaCl in 1000 mL of distilled water. This was the first step in the microplastic extraction procedure. The mixture was then agitated with a magnetic stirrer on a heated plate at 50 °C for 15 min to guarantee homogeneity, and density separation was performed to exclude inorganic particles. To avoid dilution, the mixture was then moved to a separating funnel, washed with a strong salt solution (5.00 M NaCl), and allowed to settle for about a week. The microplastics remained in the solution after the sediment was drained and eliminated after it had settled (Asadi et al., 2025; Cutroneo et al., 2021; Monteiro et al., 2022). After that, 30% hydrogen peroxide (H_2_O_2_, Grade AR) was added to the sample at a 1:10 ratio to remove organic materials. The sample was then heated to 50 °C for an hour and then allowed to cool at ambient temperature for 24 h. Upon organic and inorganic separation, the sample was further prepared based on the requirements of analysis under the fluorescence microscope and µFTIR.

### Microplastic characterization

A total of 15 L of water was collected at each sample session (e.g., SF1), of which 4 L was used for comprehensive analysis. The 4-L sample was split into four 1-L samples, which were analyzed in quadruplicate (one original and three replicates) using fluorescence microscopy to ensure precise and reliable results. Similarly, for FTIR microscopy, the 4-L sample method from the initial 15-L collection was used.

#### Visual analysis using fluorescence microscopy

The Nile Red (NR) staining method was employed for the fluorescence microscopy analysis. NR (technical grade, 97% Sigma-Aldrich, Co. Germany) stock solution with a concentration of 0.05 g/L was prepared in acetone C_3_H_6_O (technical grade). The stock solution is diluted with n-hexane 95% (HPLC grade) to generate 10 mg/L of NR working solution. 0.15 ml of staining agent was added to 1 L of the water sample before sample analysis, and the mixture was left to react at room temperature for 24 h (Idehara et al., 2025). Once the reaction time was completed, the samples were filtered through vacuum filtration with a Sterile MCE membrane filter (47 mm Ø, 0.45-µm pore size) having gridded lines. After filtration, the filter paper was placed in an aluminum weight boat and stored in a glass petri dish, subsequently allowing the sample to dry at room temperature for 24 h before observation under the fluorescence microscope.

The quantity, shape, and size of the MP particles on the gridded filter paper were observed using a fluorescence microscope (Olympus BX41, Japan) outfitted with a Lumenera INFINITY3 camera and Infinity Analyze Software. The MPs were examined in green (dichroic mirror: DM500; excitation filter: BP 450–480; barrier filter: BA 515) and red (dichroic mirror: DM 570; excitation filter: BP 510–550; and barrier filter: BA 590). Images were captured at 10 × magnification, having a 200-µm scale bar. The stained MPs were able to be observed fluorescing in both colors, red and green (Maes et al., 2017). Ultrapure water samples were used as a negative control. In addition, water samples not stained with NR were imaged as background fluorescence. Average readings and selected images from the quadruplicate analyses were reported.

#### Polymer identification and count using FTIR microscope

For the µFTIR analysis, 1 L of water samples was filtered through pre-decontaminated (600 °C, 1 h) ANODISC membrane filters (Whatman Alumina Oxide, 0.2 µm, 13 mm) and stainless-steel mesh filters (20 µm, 13 mm). The filter papers were dried and kept in Petri plates that had been triple-rinsed (using distilled water) for 24 h. Micro-FTIR analysis was conducted using a Nicolet iN10 MX instrument in transmission mode with a liquid nitrogen-cooled MCT detector. The instrument settings included a wavenumber range of 4000–675 cm^−1^, 64 scans, and a spectral resolution 16 cm^−1^ and 25-μm spatial resolution. Samples were identified as polymers if they matched spectral libraries (e.g., Aldrich Polymer Library) with ≥ 80% similarity. Analysis was performed using Omnic Picta software, with each sample set analyzed in quadruplicate and average readings reported. The spectral background was obtained from a clean portion of the filter, and samples were matched against the instrument library and earlier studies. Average readings and selected images from the quadruplicate analyses were reported (Anuar et al., 2023; Faikhaw et al., 2025).

## Results and discussion

### MPs identification and quantification via fluorescence microscopy

**Fig. 2:**
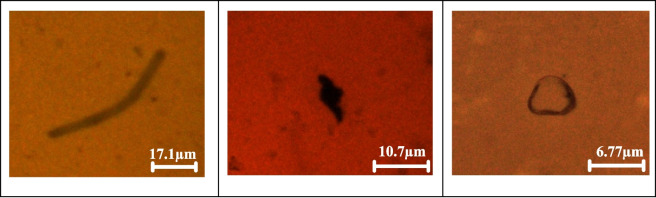
Sample images of non-fluorescence particles under a microscope. The scale bar indicates actual length of the particle in the image

MPs’ shape, particle size, and quantity of particles have been determined using fluorescence microscopy. The manual calculation was made based on the image capture and observation under fluorescence microscopy. Three water samples of Perak River water were observed from the two seasons. SF1, SF2, and SF3 are the data obtained for February 2023, and SJ1, SJ2, and SJ3 are for July 2023. The analysis of quadruplicate samples revealed an average, a total of 220 ± 43.14 and 243 ± 58.71 MPs particles/L were observed in Perak River water drawn in February and July, respectively. The mean ± SD concentrations of MPs in each sample were found to be 200 ± 27.22 particles/L (SF1), 268 ± 21.52 particles/L (SF2), and 168 ± 13.00 particles/L (SF3) in February, and 212 ± 4.93 particles/L (SJ1), 311 ± 8.19 particles/L (SJ2), and 207 ± 6.66 particles/L (SJ3) in July, respectively. It is to be noted that there were unknown particles in the water samples. 385 ± 38.58 particles/L and 589 ± 43.31 particles/L in February and July, respectively, were unable to be identified/confirmed as MPs. This is because these particles were not fluorescing when seen under the fluorescence microscopy examination (Fig. 2). Particles that were not fluorescing under the microscope are assumed to be of natural or non-plastic origin, such as clay, filled sodium silica, and alumina (Myszka et al., 2023).

**Fig. 3:**
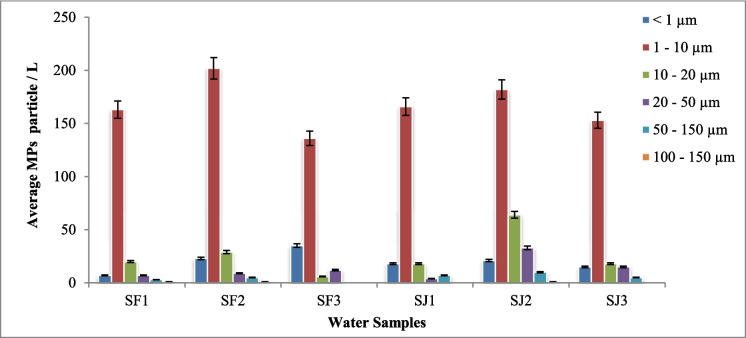
Size distribution of microplastics (MPs) in February (SF1-SF3) and July (SJ1-SJ3) water samples, analyzed using fluorescent microscopy

It is known that some of the polymers present in water samples are not reactive with Nile red (Bianco et al., 2023). There were many unknown particles as well, which could be inert materials such as silica. If compared to the Kelantan River 179.6 items/L, the average number of MP particles in the Perak River was alike (Anuar et al., 2023). However, a river’s size, flow rate, and turbidity are important factors in influencing the number of contaminants it transports. Therefore, these variables may be taken into consideration in the future as one of the main points of view that should be discussed together with other environmental considerations to assess whether there is any true argument based on the physical qualities of the river. Figure 3 shows the size distribution of particles found in February and July.

Most MP particles found were in the 1 < × < 10 µm size range, similar to findings by Martin Pivokonsky (2018), with 95% falling within this range. Nonetheless, certain research shows that the MP size distribution is biased towards smaller particles, due to the breakdown of bigger plastic waste; therefore, MP abundance increases with smaller sizes (Pivokonsky et al., 2018; Triebskorn et al., 2019). MPs with sizes > 150 µm were not detected in the water samples. This is due to the removal of larger particles by the pre-filtration of river water near the water intake point or the settling of larger particles near the water intake point. The initial size of MP particles can vary depending on their source (Jin et al., 2023). MP pollution in the Perak River could be due to municipal and industrial wastewater, agricultural run-off, human-persuaded actions such as urbanization, increased use of water resources, industrial and agricultural operations, and other natural processes such as changes in precipitation inputs, soil deterioration, and erosion. MP particles could be embrittled into different sizes and shapes by environmental conditions, for example, exposure to UV radiation and temperature fluctuations, physical forces such as abrasion and water currents, biological interactions via microbial degradation and ingestion by aquatic organisms, chemical processes, and the duration of their presence in the river environment (Gan et al., 2022). These factors collectively contribute to the size distribution of MP particles in river water, spanning from larger particles down to the nanoscale, making MP pollution a complex and dynamic issue to address. MPs are especially concerned because their bioaccumulation potential grows with the decrease in size. Numerous chemical additives are included in plastics, which also absorb organic pollutants from their surroundings. These substances are a source of chemical exposure to animals since MPs serve as carriers of other organic pollutants, which can be ingested by creatures (Wagner et al., 2014).

**Fig. 4:**
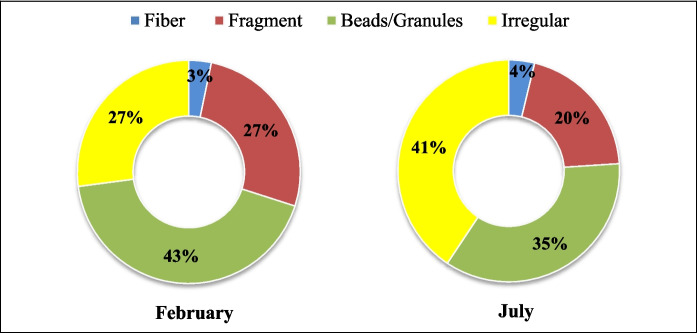
Distribution of the MPs shapes in February and July water samples

**Fig. 5:**
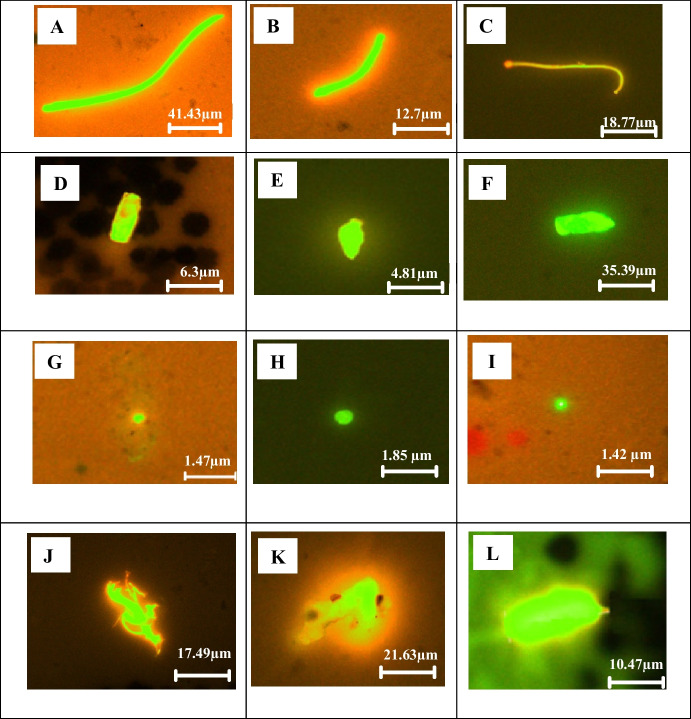
Sample images of microplastics (MPs) categorized into fiber (**A**–**C**), fragment (**D**–**F**), granules (**G**–**I**), and irregular (**J**–**L**) shapes. The particles are fluorescent due to Nile Red staining, indicating the presence of MPs, and do not represent their original color. The scale bar indicates actual length of the particle in the image

Microplastics of various shapes were detected, and their percentage occurrence is presented in Fig. 4, while Fig. 5 shows sample images of MPs’ shapes taken using fluorescence microscopy. The most common shape of MPs in the Perak River was granules, followed by fragments. However, there was also a huge percentage of irregularly shaped MPs that were difficult to identify as a standard shape.

There were also some fine particles found. Unfortunately, the shapes cannot be identified as the images are too small. MP particles were in film-like form, indicating that they may be pieces of food containers and plastic bags from consumer goods, or they could be materials from more locally focused fishing operations. A limited number of fibers were identified, similar to other Malaysian studies (Anuar et al., 2023; Liu et al., 2022; Sarijan et al., 2018). Human activities like washing clothing often release fibers into the environment, with more noticeable release patterns in greater proximity to metropolitan areas (Klein et al., 2023). Yang et al. (2019) reported that longer microfibers (> 1000 µm) were kept in the lint filter bags of a pulsator laundry machine, while shorter microfibers (500 µm) were discharged into the drainage system. MPs are smaller because bigger litter fragments decompose in the environment. The lower fiber concentrations in this work could be due to the absence of larger population centers in the distant panhandle area or upstream of the Perak River. As reported by Jin et al. (2023), the presence of granules mixed into paint or fragments of pellet MPs can be attributed to the wear and tear of coatings. Notably, most samples collected from the southern Jiangsu canal were found to have paint and coating residues (Jin et al., 2023), suggesting that the water samples from the Perak River may have granule-shaped MPs resulting from the wastewater discharge from various manufacturing industries in Perak State.

### MPs identification and quantification using FTIR microscopy

**Fig. 6:**
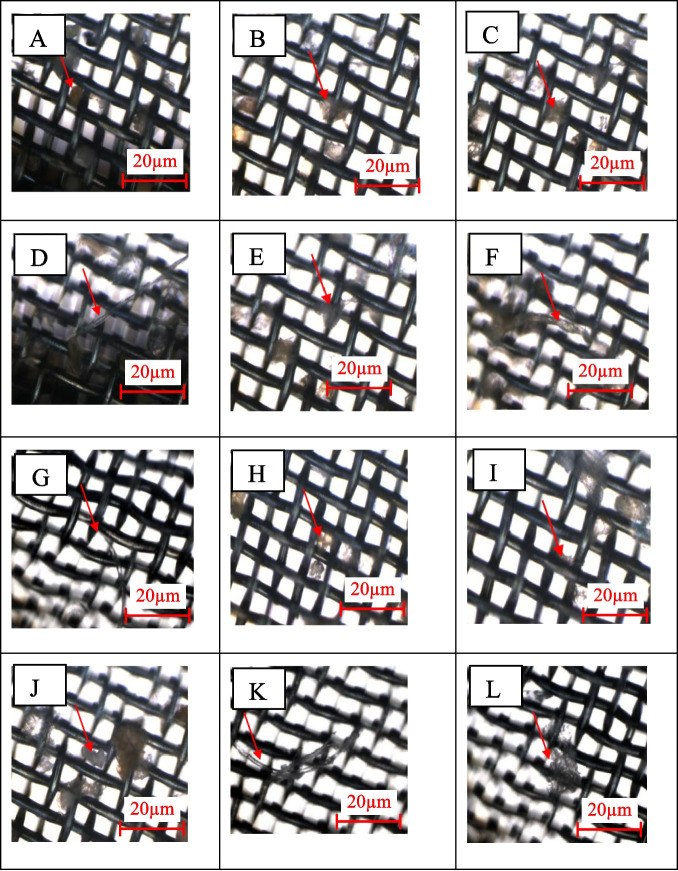
Sample images of MPs polymers captured via µFTIR: polyethylene (**A**–**C**), rayon (**D**–**F**), polyvinyl stearate (**G**, **H**), melamine–formaldehyde (**I**), polyethylene terephthalate (**J**, **K**), polypropylene (**L**). The scale bar indicates image size

FTIR microscopy identifies and characterizes microplastics by determining polymer types, chemical composition, size, and shape. In this study, 15 L of water was collected for each sample (SF1, SF2, SF3, SJ1, SJ2, SJ3), with 4 L analyzed in quadruplicate, and average readings along with selected images were reported. Figure 6 shows the image of the Anodisc filter and the particles captured on the filter. Table 2 shows the types of polymers identified in the Perak River.

**Table 2:** Types of polymers identified in the Perak River water samples using µFTIR

Types of polymers	Number of particles/L					
	February 2023			July 2023		
Water samples	SF1	SF2	SF3	SJ1	SJ2	SJ3
Ethylene propylene diene monomer (EPDM)	19.0 ± 2.0		5.0 ± 2.0		4.0 ± 2.0	4.7 ± 2.1
Melamine–formaldehyde (MF)	2.0 ± 1.0	1.3 ± 0.6	2.0 ± 1.7		3.0 ± 2.0	3.0 ± 1.0
Polyacrylamide (PAM)	1.0 ± 0.0		1.0 ± 0.0		2.0 ± 1.7	
Polyethylene (PE)	1.0 ± 0.0	16.0 ± 2.7	10 ± 2.0	9.0 ± 2.7	2.0 ± 1.0	3.3 ± 1.5
Polyethylene terephthalate (PET)		13.0 ± 2.0	7.0 ± 2.0	5.0 ± 2.0	3.0 ± 1.7	11.3 ± 2.5
Polypropylene (PP)				2.5 ± 0.7	2.0 ± 1.0	2.3 ± 1.5
Polymethyl methacrylate (PMMA)		1.0 ± 0.0				
Polystyrene (PS)	2.0 ± 1.0	2.0 ± 1.7	2.0 ± 1.0		8.0 ± 2.0	4.7 ± 1.5
Polysulfone	3.0 ± 2.0	3.0 ± 2.7				
Polyurethane (PU)	2.0 ± 1.0	2.0 ± 1.0	1.0 ± 0.00		4.0 ± 2.0	4.0 ± 1.0
Polyvinyl stearate (PVS)					4.0 ± 1.7	
Rayon	16.7 ± 2.1	19.0 ± 3.0	18.0 ± 2.7	12.0 ± 3.0	16.0 ± 3.6	16.0 ± 2.0
Total MP particles/L	46.7 ± 9.08	57.3 ± 13.6	46.0 ± 11.4	28.5 ± 8.4	48.0 ± 18.8	49.3 ± 13.2
Total unknown particles/L	172.3 ± 20.74	169.3 ± 15.50	206.0 ± 7.81	224.7 ± 24.13	192.7 ± 15.82	218.7 ± 21.7
Total MP particles/L for the month	50.0 ± 6.4			41.9 ± 11.6		
Total unknown particles/L for the month	182.5 ± 20.38			212.5 ± 16.27		

In water samples from the point of abstraction near the Perak River in Malaysia, MPs were found to have mean ± SD concentrations of 50.0 ± 6.4 particles/L in February, 41.9 ± 11.66 particles/L in July, and unknown particles 182 ± 20.38 particles/L in February and 212.5 ± 16.27 particles/L in July, as shown in Table 2. Based on the µFTIR analysis, rayon (36%), ethylene propylene diene monomer (EPDM) (20%), polyethylene (PE) (17%), and polyethylene terephthalate (PET) (13%) were the most detected polymers. Similarly, rayon and PE were recorded as high in the Kelantan Rivers (Anuar et al., 2023). The Terengganu and Sarawak, Johor River also shows similar types of MPs (Primus & Azman, 2022). Compared to the existing river water studies in Malaysia, the present study on the Perak River has found seven new types of MPs, i.e., EPDM, MF, PAM, PMMA, PU, polysulfone, and polyvinyl stearate (PVS). The chemical identification of particles using µFTIR found many particles that do not match any of the polymers in the polymer library. Other than MPs, some particles were identified as clay-filled sodium silica, alumina silicate, graphite lubricant, and road film from auto paint surfaces, and most of them were unknown particles due to lack of spectral library.

Shapes identified in this study were fiber, fragment, and irregular, with a size range of 40–160 µm. As shown in Fig. 6, polymers identified as rayon and PET had fiber shapes. On the other hand, PE and PVS fragments and other polymers have irregular shapes. Similar to the fluorescence microscopy examination, many unknown particles or non-polymer particles were identified via µFTIR. Further characterization is needed to identify the particle components and their toxicity (Liu et al., 2024). Luo et al. (2019) also reported that 800 items were recovered, and only 285 (32.1%) were validated as MPs, with the remaining particles categorized as “unknown.” The final number of MPs in their study was recalculated by removing the unverified microplastics (Luo et al., 2019).

It is noted that rayon is more commonly detected in Malaysian rivers. Rayon is a semisynthetic cellulose-based polymer, commonly utilized in artificial fabrics, products for personal hygiene, and the fishing industry, such as fishing nets (Anuar, 2020; Chen, 2023; Yang Hwi et al., 2020). Rayon fibers can be shed from textiles during various stages of their lifecycle, such as washing, wear, or disposal, and enter waterways via runoff. Reports from other countries, such as China, also identified the existence of rayon in freshwaters (Cao et al., 2021; Liu et al., 2019). Therefore, the study area’s high rayon concentration may be the consequence of indirect input via wastewater discharge from 10 populated towns, including major areas such as Gerik, Kuala Kangsar, Teluk Intan, and Bagan Datoh, as well as from other industries along the Perak River basin (Salam et al., 2020; Zeshan et al., 2021). Like other MPs, rayon fibers can have adverse effects on aquatic life (Jiang et al., 2024), as they are small enough to be ingested by aquatic organisms, potentially harming them and entering the food chain. Mitigating this pollution involves better textile waste management, adopting eco-friendly textiles, and addressing the sources of microfiber shedding to reduce their entry into river systems.

Common plastics like PE and PP have densities similar to water, which allow them to float or stay suspended in water. They are often found in rivers because of their durability. Also, these polymers are more likely to infiltrate streams through a variety of channels, including litter, runoff (Anuar et al., 2023). According to studies conducted in Malaysia, Thailand, Indonesia, Vietnam, the Philippines, and Cambodia, PE, PP, and PET were derived from major sources such as household and industrial plastic waste, agricultural runoff, wastewater treatment plants, and the shipping and fishing industries (Finnegan et al., 2021; Irfan et al., 2024; Mat et al., 2022). Twenty-four percent of the major composition of MPs observed in freshwater globally was identified as PP material (Chen et al., 2021; Li et al., 2020). In the present study, PP was found only in July samples in the Perak River, indicating a possible lower usage of PP in the drainage basin. The washout of PP during the rainy season (February), along with storm water runoff, can be ignored as it does not show any signature relation to the presence of PP.

Researchers use the polymer hazard index (PHI) and pollution load index (PLI) to assess ecological risks of polymers. High-risk polymers such as polyvinyl chloride (PVC) and polyurethane (PU) have hazard scores of 10,001 and 13,844. Other polymers, including polyamide (PA), polystyrene (PS), polyethylene (PE), polyethylene terephthalate (PETE), and polypropylene (PP), show lower hazard scores (Hossain et al., 2023; Yang et al., 2023). Interestingly, the PLI of PE could be higher in certain regions due to its abundance (Wei et al., 2025), particularly in the Perak River. However, there is no available data for the Perak River to calculate the PLI and PHI for microplastics in this study. As such, the specific ecological risks of MPs in this area remain uncertain, highlighting the need for further research to measure the pollution load and hazard index of all 12 of the polymers found in this study (Choudhary et al., 2025).

### Comparison of detection methods

**Fig. 7:**
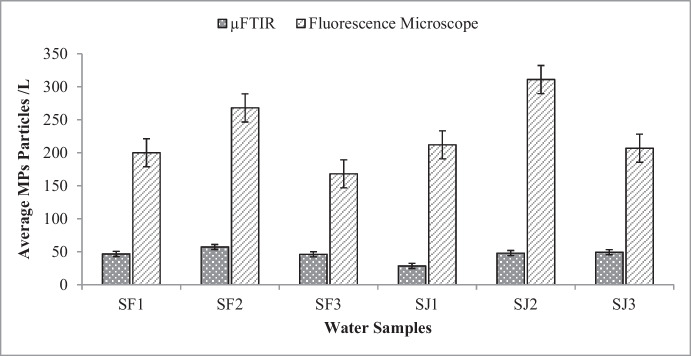
Comparison of MPs abundance by µFTIR and fluorescence microscopy techniques

This study compared fluorescence microscopy and µFTIR for microplastic detection in Perak River water samples. Fluorescence microscopy identified more microplastics, leveraging their fluorescence properties under specific wavelengths. In contrast, micro-FTIR analysis, using a Nicolet iN10 MX instrument with OMNIC Picta software, characterized microplastic particles on filter membranes. The differences in detection are illustrated in Fig. 7. Fluorescence microscopy facilitates the visualization and quantification of MPs and has comparatively cheap acquisition costs. This method has several unique advantages, including high sensitivity and the ability to detect MPs at low concentrations, which makes it a valuable tool for identifying microplastics in environmental samples. However, a major drawback is that it may not be able to distinguish between different types of plastics and can occasionally produce false positives. In contrast, µFTIR offers high chemical specificity, which allows for the accurate identification of different polymer types, but it has significant disadvantages, including high equipment costs and complicated sample preparation requirements. Despite these issues, µFTIR offers detailed information about the chemical composition of MPs, making it a powerful tool for characterizing microplastics in environmental samples (Guzman et al., 2022; Shim et al., 2016; Sturm et al., 2021).

However, fluorescence microscopy could only be used to identify fluorescent polymers. It is known that some polymers do not have the properties of being fluorescent in Nile Red, and some random particles could have the ability to be fluorescent in Nile Red (Bianco et al., 2023; ‌Nam et al., 2024). Thus, the results obtained might have some inaccuracy. Moreover, this technique does not provide any information on chemical composition. µFTIR is more robust in identifying polymer types since each particle has a unique spectrum that enables the separation of plastics from other organic and inorganic particles; also, µFTIR has also been used to enhance the precision of MP quantification. This method is limited by its reliance on the µFTIR software library, which can only identify polymers included in its database. Particles that are not in the library may go undetected. Also, due to the instrument’s threshold, it can only detect MPs’ sizes limited to *X* > 20 μm(Gupta et al., 2024). Expanding the library with new reference spectra can improve identification. This was evident when many unknown particles and a smaller number of particles were identified via µFTIR in this study.

These constraints limit the ability to determine the sources, fate, and potential short- and long-term environmental repercussions of these MPs, as well as advise policymakers on how to control MPs’ pollution. The choice between these techniques depends on the availability of equipment, specific analytical goals, and the nature of the MPs under investigation. Compared to other reports from Malaysian rivers, only Anuar (2023) has used µFTIR to identify MPs in river water. The previous study revealed that 14 types of polymers were identified in the Kelantan River and 20 types of polymers in the Langat River, respectively. The majority of the polymer particles belong to the PE, PP, and rayon groups. The findings were almost similar to the current findings, and it was highlighted that smaller polymers were unable to be detected by µFTIR identification. There was no report using Nile red and fluorescence microscopy techniques for MP identification in Malaysian river water. The other studies used methods such as stereomicroscopes, binocular microscopes, scanning electron microscope–energy-dispersive x-ray spectroscopy (SEM–EDS), pyrolysis-GC/MS, and attenuated total reflectance Fourier infrared spectroscopy (ATR-FTIR) (Anuar et al., 2023; Karing et al., 2023; Primus & Azman, 2022; Yang et al., 2020; Pariatamby et al., 2020; Sarijan et al., 2018).

Other than Malaysia, ASEAN member countries such as Cambodia, Indonesia, the Philippines, and Thailand also reported studies on MPs in river water (Irfan et al., 2024; Nakano et al., 2025). However, none were reported on the river water point of abstraction for the water treatment plant. Most of these countries reported polymer types by ATR-FTIR, µFTIR, and Raman spectroscopy; only one study in Indonesia reported µFTIR spectroscopy, and the study quantified 113.33 particles/m^3^ in the Cisadane River. The chemical composition of 122 detected particles (30.35% of total recovered particles) was determined using µFTIR spectroscopy, and 11 MPs polymer types were identified (Sulistyowati et al., 2022).

In comparison to other global studies, there were also no reports on the river water point of abstraction for water treatment plants. Recently, there were findings from Japan and China on the Tsurumi River and the Wen-Rui Tang River, respectively, using µFTIR and fluorescence microscopy. Wang et al. (2018) reported that MPs smaller than 20 μm were challenging to identify with µFTIR; hence, it was considered for particles in the 20–5000 μm size range. According to Shruti et al. (2022), fluorescence microscopy and µFTIR spectroscopy yield comparable results in MP quantification. Fluorescence microscopy, particularly with Nile Red staining, proves to be a valuable method for initial screening due to its rapidity and effectiveness. However, potential drawbacks include false positives from non-plastic particles and inconsistent fluorescence patterns based on polymer characteristics. To guarantee precision, µFTIR analysis is advised for confirming the chemical makeup of suspected microplastics. By integrating both methods, researchers can capitalize on their respective strengths: the swift detection capability of fluorescence microscopy and the accurate chemical identification of µFTIR spectroscopy. This combined approach boosts the dependability of microplastic detection and quantification.

The results of this study show that the majority of MPs found in the sample are in the form of granules, fragments, and irregular forms. This is a troubling discovery since the environmental relevance of MPs in the form of granules and irregulars poses a danger to ecosystem health. Small creatures can easily swallow granular MPs, resulting in bioaccumulation and physical damage. Furthermore, unevenly shaped MPs can entangle and choke creatures while providing a surface for algae to grow, resulting in biofouling. Both forms of MPs can leach pollutants, change habitats, and disrupt food chains, posing a danger to ecosystem function and human health. These results highlight the need for more studies on the effects of MPs on the environment and human health. Mitigating microplastic pollution is crucial for protecting ecosystem resilience.

### Source of MPs in Perak River

Human activities, including industrial, agricultural, and urban practices, significantly contribute to microplastic pollution in rivers. The Perak River, in particular, is impacted by a range of activities, such as sand mining, shipping, steel production, and manufacturing, along with non-point sources like untreated wastewater and urban runoff. One of the studies has highlighted that trace metals in the Perak River could also be an agent to carry microplastics in aquatic environments (Salam et al., 2020). The abundance of MPs in freshwater ecosystems varies depending on geographical and pollution input sources (Gunaalan et al., 2023; Padha et al., 2025). In Malaysia, while some progress has been made, data on microplastic distribution across different aquatic systems—rivers, tributaries, estuaries, and coastal areas—remains limited. The Perak River is lined with heavy industries, including those involved in steel production, plastic waste recovery, and wastewater treatment, all of which contribute to its microplastic load (Ahmad et al., 2017; Salam et al., 2019a, 2019b; Salam et al., 2019a, 2019b; Zeshan et al., 2021).

The Perak State experiences a tropical rainforest climate with high rainfall throughout the year compared to other states in western Malaysia, although there are seasonal variations, with February being part of the wet season and July falling in the drier season (Malaysian Meteorological Department, 2023; Perak State Irrigation and Drainage Department, 2022). Despite this difference in rainfall pattern, the number of MP particles is the same in the Perak River at Parit Water Intake facility (4°29′10.4″N, 100°54′50.2″E). The consistent presence of microplastics in the Perak River, despite seasonal rainfall variations, suggests that industrial activities, plastic waste, and urban runoff are continuous contributors. This highlights that industrial operations and urbanization within the catchment area are the primary drivers of microplastic contamination. The Perak River’s water quality has further deteriorated due to rapid urbanization and industrial development, increasing ecological risks and the potential for microplastic contamination of aquatic life, including fish. The concentration of microplastics in the river is dependent on industrial and urban inputs (Kataoka et al., 2019; Verma et al., 2025).

## Conclusion

This study reveals severe microplastic (MP) contamination in the Perak River, with concentrations remaining relatively stable during both the wet and dry seasons. The particles were irregularly shaped and varied in size from 1 to 10 µm, largely consisting of rayon and polyethylene (PE). FTIR and fluorescence microscopy revealed significant numbers of MPs. In February, the mean ± SD concentrations ranged from 168 ± 13.00 to 268 ± 21.52 particles/L. In July, values ranged from 207 ± 6.66 to 311 ± 8.19 particles/L. The patterns are similar to those seen in other Malaysian rivers. Sungai Dungun had MP levels ranging from 38.7 to 300.8 items/m^3^, whereas Kelantan had 179.6 items/L and Kemena had 60–128 items/L. All locations shared polymers like rayon, PP, PET, and PE. Poor handling of plastic garbage is the cause of similar problems throughout ASEAN, such as in Thailand’s Chao Phraya River. These results highlight the necessity of stricter regulations governing plastics, improved waste management systems, producer accountability, and public education. To safeguard drinking water and promote sustainable river management, ongoing observation and sophisticated analysis are crucial.

## Data Availability

No datasets were generated or analysed during the current study.
